# Scion organ removal alters hormone levels and gene expression associated with adventitious root development in grafted watermelon seedlings

**DOI:** 10.1080/15592324.2025.2556300

**Published:** 2025-09-12

**Authors:** Ce Song, Yi Huang, Chenchen Wu, Baoming Tian, Xuanjie Shi, Guoquan Mi, Yancai Jing, Yanling Tang, Zuojing Wang, Lili Niu, Tengqi Wang, Gongyao Shi, Kai Ma

**Affiliations:** a Institute of Vegetables, Henan Academy of Agricultural Sciences, Graduate T&R Base of Zhengzhou University, Zhengzhou, China; b School of Agricultural Sciences, Zhengzhou University, Zhengzhou, China

**Keywords:** Adventitious root, watermelon, scion, transcriptome, phytohormone

## Abstract

Adventitious roots (ARs) are crucial for grafted watermelon seedlings, playing vital roles in nutrient absorption, stress resistance, and grafting efficacy. However, the way in which scions regulate endogenous hormones to influence AR formation remains poorly understood. In this study, we constructed watermelon seedlings (WP) using “HXX” as the scion and “Tie Zhen No. 3” as the rootstock. Scion cotyledons removal (WP-1) significantly promoted AR development. In contrast, true leaf removal (WP-2) had minimal effect, while simultaneous removal of both (WP-3) elicited intermediate responses. Endogenous hormone dynamics showed that WP-1 maintained progressively increasing indole-3-acetic acid (IAA) with lower abscisic acid (ABA) and jasmonic acid (JA) levels, whereas both WP-2 and WP-3 exhibited divergent hormonal profiles in ARs during later development stages. Transcriptome sequencing revealed that differentially expressed genes (DEGs) are enriched in various hormone signaling pathways. On the fourth day, when the number of differential genes was the highest, the DEGs significantly expressed in all three treatment groups were enriched in the activation signaling pathways and responses of JA, auxin, ethylene, and cytokinins. Transcription factors such as bHLH, ERF, MYB, and NAC were significantly expressed during the development of ARs, playing a key regulatory role. The Kyoto Encyclopedia of Genes and Genomes (KEGG) analysis identified 82 DEGs across five hormone signal transduction pathways. The weighted gene co-expression network analysis (WGCNA) identified modules positively correlated with AR hormones, highlighting hub genes such as ethylene transcription factors (CRF4, ABR1, ERF054, ERF098), auxin response factors (SAUR21 and SAUR32), and other regulators (CSA, HSP, bHLH93, ZAT5, ZAT13, NAC, MYB, and C3H). These findings provide preliminary evidence of the scion's regulatory role in AR development through hormones, offering a foundation for improving watermelon grafting practices.

## Introduction

Grafting technology is an important production measure used to overcome continuous cropping obstacles and soil-borne diseases and to increase the yield of current watermelon production. Selecting suitable rootstocks and effective grafting methods is the foundation for cultivating strong watermelon seedlings^1^; in this regard, pumpkin remains the best rootstock for watermelon grafting. Grafting transforms cucurbit plants by enhancing their root systems, which leads to a marked improvement in rootstock vitality, resulting in significantly better water and nutrient uptake. Consequently, these plants exhibit increased photosynthetic efficiency due to the improved resource supply. In addition, grafting reinforces their antioxidant defense mechanisms and increases their capacity for long-distance transport.^2^ Roots are crucial for grafted plants, anchoring them in the soil and facilitating water and nutrient acquisition. While AR formation is vital for the success of double-root-cut grafting, knowledge regarding AR development specifically at the rootstock stem in soil conditions remains limited.

ARs, by definition, originate from non-root tissues like stems, leaves, or calluses.^3^^,^^4^ The scion exerts a significant influence on AR formation in the rootstock. It acts as a source of vital signals, providing phytohormones like auxin and photosynthates that are transported to the rootstock to stimulate AR development, particularly at the stem base. Furthermore, by orchestrating AR formation in the rootstock through hormonal, nutritional, and mobile signals, the scion influences AR characteristics like length, root surface area, and biomass. This dynamic regulation ensures plant survival and growth when roots are lost, underscoring the scion's critical regulatory role in this grafting method.^5-8^ Plant hormones are the main signaling molecules that regulate the formation of ARs, and their content in plant metabolic products is very low. They can be transported from their synthesis sites to other locations to exert regulatory effects. For example, hormones related to plant growth, such as auxin, ABA, JA, cytokinins, gibberellins, strigolactones (SLs), and ethylene, can promote or inhibit the formation of ARs in plants at different concentrations.^9^^,^^10^

The levels of plant hormones and the genes and transcription factors associated with plant hormone signaling pathways play an indispensable role in the formation and development of ARs.^11^^,^^12^ Auxin is a growth regulator that plays a central role in the rooting process. Most other plant hormones need to interact with auxin to exert regulatory effects on the growth of ARs. Auxin can regulate the formation of ARs by inducing cell division, elongation, and differentiation.^13^ Far-red light can significantly induce the expression of auxin response proteins (IAA11, IAA17, and AUX28), small auxin RNA genes (*SAUR20* and *SAUR50*), and auxin efflux transporters (*PIN3*) in watermelon. It also significantly increases the expression of phytochrome-interacting factors (PIFs), such as *PIF1*, *PIF3*, and *PIF7*. These genes may work together under far-red light treatment to activate auxin-related pathways to regulate the formation of ARs.^14^ The exogenous application of GR24 (an analog of the strigolactone) promotes the development of ARs in melons. GR24 treatment combined with IAA further increased the number, length, and surface area of ARs. Notably, GR24 + IAA treatment further elevates auxin, GA, and zeatin levels. In contrast, the ABA content significantly decreases.^15^ JA interacts with auxin and other plant hormones through complex crosstalk pathways during the development of ARs, enhancing auxin synthesis during the induction phase, and promoting AR formation. Low concentrations of methyl jasmonate promote AR development, while high concentrations inhibit it. JA can also interfere with ethylene signaling to promote the occurrence of auxin-induced ARs in Arabidopsis seedling skotomorphogenesis.^16^^,^^17^ Ethylene is considered a stimulator of the early induction and late formation of ARs, and JA can interact with it to regulate the occurrence and quantity of ARs. Ethylene can exhibit a promoting effect on the AR formation process under different conditions, which has been confirmed in crops such as tomato and cucumber. However, some studies indicate that ethylene only plays a negative role during the late AR induction phase and the formation of lateral roots, suggesting that ethylene has two completely opposite effects on AR development.^18^^,^^19^ In addition to auxin, JA, ethylene, ABA, salicylic acid, brassinosteroids, and other hormones can also play various roles in the occurrence of ARs in plants. These findings indicate that there are complex interactions between various hormones during the formation of ARs, but auxin plays the most important role, as it can interact with almost all hormones. The mechanisms of how these hormones interact still require further research.^20-22^

Transcriptome, microarray, and genomic sequencing, among other omics technologies, have been widely applied in recent years to study AR formation in various species, including melon,^14^^,^^15^ poplar,^23^ and apple.^24-26^ Most of these sequencing technologies aim to identify key genes and metabolic pathways that regulate the development and formation of ARs. Weighted gene co-expression network analysis (WGCNA), based on high-throughput microarray or RNA-Seq data, identifies co-expressed gene modules and explores connections between gene networks and target phenotypes at the transcriptional level.^27^^,^^28^ As an efficient and accurate bioinformatics method, this algorithm effectively integrates with transcriptomics to explore the molecular mechanisms of endogenous hormone regulation in AR formation in depth.^29^

In this study, the treatment of scion cotyledons and true leaves promoted the development of ARs and the increase in three endogenous hormone levels to varying degrees. Transcriptomic analysis revealed significant enrichment of DEGs in various hormone-related biological processes, including auxin, jasmonic acid, ethylene, and cytokinins. KEGG pathways annotated multiple differential genes that are significantly expressed in different hormone signaling pathways. A further WGCNA identified key modules positively correlated with the three hormones, where the hub genes included several candidate genes related to auxin and ethylene, as well as various key transcription factors. These are important components that play regulatory roles in the development of ARs and are focal points for future research. Taken together, this study provides a molecular-level explanation for the limitation of AR development in grafted seedlings by hormonal regulation, contributing to the efficient and sustainable development of watermelon production.

## Materials and methods

### Plant materials and growth conditions

This study utilized “HXX” watermelon scions grafted onto “Tie Zhen No. 3” pumpkin rootstocks. Seed treatment commenced with stirring the seeds in 55 °C water for 10 min to enhance hydration, followed by a 2-h soak in a diluted fludioxonil-metalaxyl fungicide solution (0.104 g/l active ingredient). Subsequently, seeds underwent surface sterilization in 0.1% potassium permanganate for 2 h, rinsing, and then drying. Prior to sowing, seeds were soaked in clean water for 2 h and briefly machine-dried. Treated seeds were germinated in moist gauze at 30 °C for approximately 12 h until radicle emergence. Sowing was conducted in 40-cell trays (two seeds per cell) covered with 0.5 cm of vermiculite, and kept warm and humid under a plastic film, which was removed after seedling emergence. Seedlings were cultivated in a greenhouse. “Paste grafting” was employed, involving a 30-Degree cut to excise the rootstock growth point, true leaves, and one cotyledon, and a matching 30-Degree scion hypocotyl cut below the cotyledons. The scion and rootstock were joined, secured with clips, and the rootstock's remaining cotyledon was removed, leaving a 5 cm hypocotyl. The graft union was positioned ~3 cm above the substrate. Grafting clips were removed after 10−15 d. Treatments consisted of single plants in 40-cell trays (120 plants/treatment) maintained at 85%−90% humidity to promote adventitious rooting. Three biological replicates of each material were sampled on Day 2, 4, and 6 to measure the AR parameters, yielding sufficient material for analysis.

### Determination of endogenous hormone content

The measured endogenous levels of indole−3-acetic acid (IAA), abscisic acid (ABA), and jasmonic acid (JA) in the tissue samples were collected from a region extending approximately 3 cm upwards from the base of the ARs along the rootstock hypocotyl, encompassing all ARs. The scion hormone content was determined using the whole scion. Sampling was conducted on Day 2, 4, and 6 post-grafting. The NM-EL0887P ELISA kit (Norminkoda, Wuhan, China) was employed for double-antibody sandwich ELISA detection. Tissue samples were frozen in liquid nitrogen, homogenized in pH 7.4 phosphate-buffered saline, and centrifuged (4 °C, 10,000 g, 15 min) to collect the supernatant. The samples were diluted 5-fold prior to analysis. The assay involved incubation at 37 °C, washing, the addition of an HRP-conjugated detection antibody, TMB (3,3′,5,5′-tetramethylbenzidine) color development, and OD measurement at 450 nm. Hormone concentrations were determined from their respective quadratic standard curves by applying the corrected sample absorbance (ΔOD) to the regression equation. The final content was then calculated by normalizing this result for sample weight, volume, and dilution.

### RNA sequencing and data processing

Samples were 3 cm rootstock segments taken upwards from the AR base. For each treatment group at each time point (Day 2, 4, and 6), three biological replicates were prepared. All collected samples were flash-frozen in liquid nitrogen for RNA extraction. Total RNA was extracted using TRIzol reagent (Invitrogen), with DNase I (TaKara) treatment used to remove genomic DNA. RNA quality was assessed (Agilent 2100 Bioanalyzer, NanoDrop ND-2000), and only high-quality RNA was used for library construction. RNA-seq libraries were prepared from 1 μg of the total RNA using the TruSeq RNA Sample Preparation Kit (Illumina). Briefly, mRNA was enriched via poly-A selection and fragmented. Double-stranded cDNA was synthesized (SuperScript Kit, Invitrogen) with random hexamer primers. Libraries were size-selected (~300 bp), PCR-amplified (Phusion DNA polymerase, NEB, 15 cycles), and quantified (TBS380). Paired-end sequencing (2 × 150 bp) was performed on the Illumina HiSeq Xten/NovaSeq 6000 platform. Raw reads were quality-controlled (SeqPrep, Sickle), and clean reads were aligned to the reference genome using HISAT2 (orientation mode).^30^ Transcript assembly was conducted using StringTie (reference-based).^31^ The RNA-seq data are summarized in Data S1, and the raw data can be accessed at PRJNA1202060.

### Analysis of differential gene expression and functional pathway enrichment

Gene expression levels were quantified as transcripts per million (TPM) using RSEM.^32^ DEGs were determined using DESeq2,^33^ with significance defined by a |log2-fold change| > 1 and a Benjamini‒Hochberg adjusted *p*-value (*Q*-value) ≤ 0.05. To investigate the functional roles of the DEGs, GO and KEGG enrichment analyses were performed. GO enrichment analysis was carried out using Goatools, and KEGG pathway enrichment was performed using KOBAS.^34^ Enriched GO terms and KEGG pathways were considered significant at a Bonferroni-corrected *p*-value ≤ 0.05.

### Weighted gene co-expression network analysis

WGCNA was performed using IAA, ABA, and JA levels as traits. A weighted correlation network was constructed by increasing the gene correlation matrix to power (*N*) to approximate a scale-free network. Hierarchical clustering was then applied to this weighted network to identify modules of highly interconnected genes. These modules, represented as branches and assigned distinct colors in the dendrogram, represent groups of genes with similar expression profiles. Within each module, genes exhibiting high intramodular connectivity were considered key drivers. For visualization and detailed examination, the top 30 most connected genes within the selected modules were extracted to generate heatmaps and expression bar plots. Module‒trait relationships were assessed by correlating module eigengenes with hormone traits to identify biologically relevant modules.

### RNA extraction and quantitative real-time PCR validation

Total RNA was extracted from the hypocotyl segment above the root base (covering all ARs within this region) using the CTAB method. RNA concentration and purity were then determined using a Nanodrop 2000 spectrophotometer. Subsequently, a reverse transcription reaction was performed using the SweScript All-in-One RT SuperMix for qPCR kit, with the reaction conditions set at 25 °C for 5 min, 42 °C for 30 min, and 85 °C for 5 s. The quantitative PCR reaction system included 2× Universal Blue SYBR Green qPCR Master Mix, gene-specific primers, and cDNA templates, conducted on the Bio-Rad CFX Connect platform. The reaction program comprised 30 s of pre-denaturation at 95 °C, 15 s of denaturation at 95 °C, and 40 cycles of annealing/extension at 60 °C for 30 s, followed by a melt curve analysis. The relative expression levels of genes were calculated using the ΔΔCT method, where A = CT (target gene, sample to be tested) − CT (internal control gene, sample to be tested), B = CT (target gene, control sample) − CT (internal control gene, control sample), K = A−B, and the expression fold change = 2^−^^K^. The qRT‒PCR data were normalized to housekeeping genes, particularly actin, prior to comparisons between various ecotypes. All the primers employed for qRT‒PCR analyses are detailed in Table S3.

## Results

### Morphological changes and endogenous hormone response during AR formation

**Figure 1.**
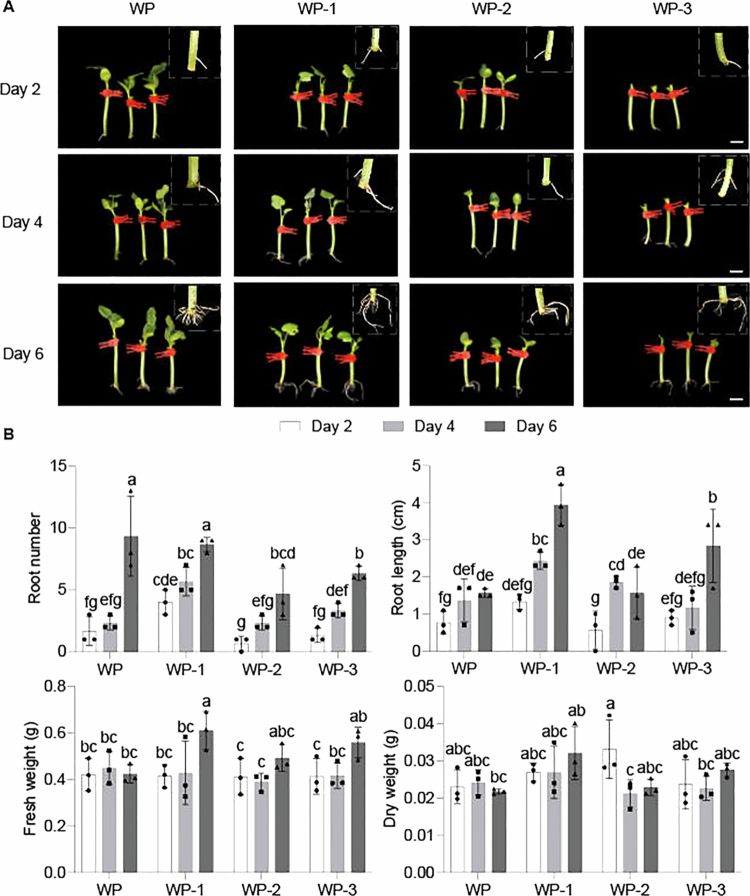
Phenotype growth parameters of grafted seedlings at different stages. (A) Phenotype of grafted seedlings (taking "HXX" watermelon as scion and "Tie Zhen No. 3" pumpkin as rootstock) on Days 2, 4, and 6. WP sample: Normal grafted seedlings without any treatment; WP-1 sample: With cotyledon of scion removed; WP-2 sample: True leaves of the scion removed; WP-3 sample: Cotyledon and true leaves of scion removed. (B) Graphs showing root number, root length, fresh weight, and dry weight. Error bars indicate standard deviations across three biological replicates. Different letters within different days indicate statistically significant differences based on one-way ANOVA. The dots represent biological replicates. Scale bar indicates 1 cm.

To explore the impact of the scion on the ARs of grafted seedlings, we constructed grafted watermelon seedlings (WP sample) using “HXX” as the scion and “Tie Zhen No. 3” as the rootstock, while performing treatments on the scion including the removal of cotyledons (WP-1 sample), the removal of true leaves (WP-2 sample), and the removal of both cotyledons and true leaves (WP-3 sample) (Figure 1A), followed by a statistical analysis of the physiological indicators of the grafted seedlings. The number and length of roots in the WP-1 sample consistently showed a significant increase during the growth process (from Day 2 to Day 6) when compared to the WP, WP-2, and WP-3 samples. In terms of these two physiological indicators, the WP-3 sample ranked between the WP-1 and WP-2 samples, with more numerous and longer roots than those in the WP-2 sample but fewer and shorter roots than those in the WP-1 sample (Figure 1B). The growth status of WP closely mirrored that of WP-2 during this period, except for the root number on the sixth day. Regarding both the fresh and dry weights, the results reflected a similar trend to that observed for root number and length. The fresh and dry weights of WP-3 on the sixth day were intermediate between those of WP-1 and WP-2, while WP exhibited almost no change during this process (Figure 1B). Overall, the removal of cotyledons from the scion can effectively promote the growth and development of ARs. While the simultaneous removal of both cotyledons and true leaves can also weakly promote AR development, the effect is markedly inferior to that achieved via the sole removal of cotyledons, indicating that true leaves exert minimal influence on their growth. This suggests a possible antagonistic relationship between cotyledons and true leaves, with cotyledons having a greater influence on the development of ARs than true leaves.

**Figure 2.**
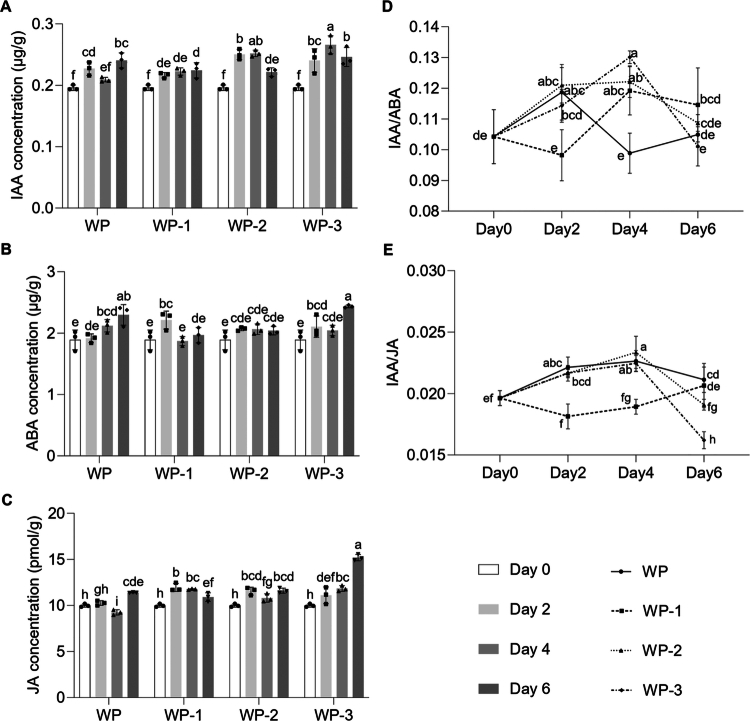
Changes in the endogenous hormone contents in ARs of rootstock at different stages. (A–C) Changes in endogenous content of IAA, ABA, and JA in the four phenotypes on Days 2, 4, and 6. (D and E) Temporal dynamics of phytohormone ratios IAA to ABA and JA are shown from Day 0 to Day 6 post-grafting. Error bars indicate standard deviations across three biological replicates. Different letters within different days indicate statistically significant differences based on one-way ANOVA. The dots represent biological replicates.

The dynamics of endogenous hormones within the ARs of the rootstock were quantified across different treatments from Day 0 to 6 post-grafting (Figure 2). Generally, IAA concentrations increased over time in most groups, with the WP-1 treatment exhibiting continuously increasing IAA levels (Figure 2A). Conversely, the ABA and JA levels in the ARs showed distinct patterns: WP-1 consistently demonstrated lower concentrations of both ABA (Figure 2B) and JA (Figure 2C) at Day 4 and 6, while WP-3 tended to accumulate the highest levels of these inhibitory hormones, particularly at later time points. The analysis of the hormone ratios revealed significant fluctuations over time (Figure 2D,E). The IAA/ABA and IAA/JA ratios in the WP-1 group increased substantially to reach peak levels at Day 4 and Day 6, consistent with its superior AR growth phenotype, which was observed later. The WP-2 and WP-3 groups exhibited variable ratio dynamics; although they showed an initial peak at Day 2, their ratios in the later stages (Days 4 and 6) are likely more reflective of established growth differences. Collectively, these results indicate that the WP-1 treatment correlated with a distinct hormonal profile in the developing rootstock ARs, which is characterized by continuously elevated IAA, relatively low ABA and JA, and consequently, significantly higher IAA/ABA and IAA/JA ratios, especially during the later stages of measurement.

To assess the role of the scion in controlling hormone biosynthesis and signaling toward the rootstock during AR development, we quantified the endogenous hormone levels and ratios within the scion tissues (Figure S1). Specifically, the WP-1 scions, which were associated with superior AR development, consistently maintained relatively low concentrations of IAA, ABA, and JA (Figure S1A–C) throughout the measurement period. In contrast, the WP-3 scions, linked to intermediate rooting, accumulated the highest levels of all three hormones (Figure S1A–C), particularly at later time points. Analyzing the hormone ratios within the scion revealed further critical distinctions: the WP-2 and WP-3 groups both exhibited significant fluctuations, reaching their peak on Day 2. Conversely, WP and WP-1 maintained consistently low ratios during the growth period (Figure S1D,E). Therefore, cotyledons and true leaves play a regulatory role in controlling the internal hormone levels in ARs. The potentially disparate hormone levels between the scion and rootstock suggest a complex interplay, rather than a simple direct correlation, in regulating the hormone content and ultimately influencing AR development.

### The transcriptional landscape during AR formation

**Figure 3.**
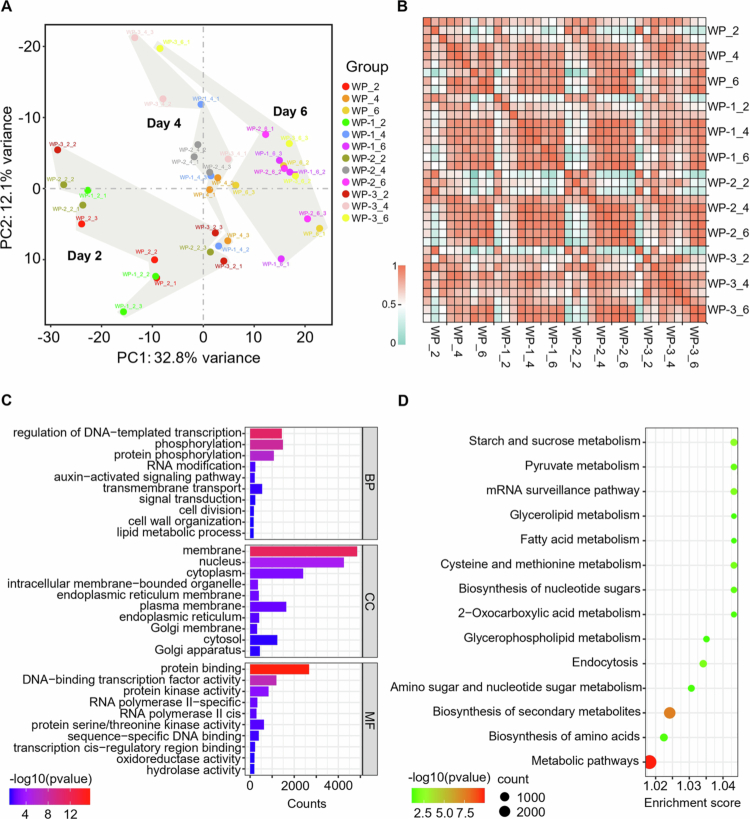
Comprehensive display and functional and pathway analysis of the transcriptome data. (A) Principal component analysis (PCA) performed using RNA deep sequencing data from WP, WP-1, WP-2, and WP-3. (B) Correlation analysis between samples. (C) Total genes from annotated databases subjected to Gene Ontology (GO) enrichment analysis. The horizontal bars represent the counts for biological processes (BP), cell compartments (CC) and molecular functions (MF), while the blue-red color bar indicates the range of log-transformed *p*-values. (D) Kyoto Encyclopedia of Genes and Genomes (KEGG) pathway enrichment analysis of total genes. The size of the bubbles corresponds to the number of genes involved, while the green-red color bar indicates the range of log-transformed *p*-values.

The scion leaves can affect the endogenous hormone levels. We subsequently explored the pathways and target genes related to hormone changes regulating AR development through transcriptome sequencing. A total of 36 samples were sequenced, yielding 1570.68 Gb of clean data, with the effective data volume for each sample ranging from 55.72 to 83.86 Gb and the Q30 base distribution ranging between 97.55% and 98.18%. The average GC content was 45.24% (Table S1). By aligning the reads to the reference genome, we obtained the genomic alignment status for each sample, with alignment rates ranging from 84.05% to 88.17% (Table S2). The PCA results showed that PC1 and PC2 explained 32.8% and 12.1% of the variation in gene expression among all of the samples collected on different days, respectively (Figure 3A). The correlation analysis indicated a strong correlation among the sample repetitions (Figure 3B).

After mapping the clean reads to the *Cucurbita moschata* genome, a total of 32,462 transcripts were finally obtained (Data S1). The results of the Gene Ontology (GO) analysis showed that these genes are significantly enriched in multiple biological processes such as transcription, protein phosphorylation, the auxin-activated signaling pathway, and signal transduction. Most genes are primarily concentrated in the cellular membrane, cytoplasm, nucleus, and intermembrane regions. Similarly, the molecular function results indicated associations with protein binding, transcription factor activity, protein kinase activity, and RNA enzyme activity (Figure 3C). The combined enrichment results reflect that the gene set performs key molecular activities at specific locations within the cell to drive fundamental biological processes. The enrichment in auxin-activated signaling pathways involves the binding of auxin to receptors on the cell membrane, indicating that our gene set participates in auxin-mediated responses.

We identified several metabolic and signaling pathways significantly associated with the formation of ARs through KEGG pathway enrichment analysis. These pathways include starch and sucrose metabolism, pyruvate metabolism, glycerolipid and fatty acid metabolism, amino acid and ribose biosynthesis, mRNA surveillance, endocytosis, and secondary metabolite biosynthesis (Figure 3D). These processes collectively provide the necessary energy, molecular basis, and cellular mechanisms for the development of ARs. The metabolism of carbohydrates and fatty acids provides energy for cell division and growth, while amino acid and ribose biosynthesis ensures a sufficient supply of proteins and nucleic acids.^35^ Additionally, mRNA surveillance and endocytosis play critical roles in cellular signal transduction and gene expression regulation, while secondary metabolites are involved in plant defense and adaptation to environmental changes.^36^^,^^37^ In summary, these enriched pathways reveal the complex molecular and metabolic networks involved in the formation of ARs, emphasizing the importance of the coordinated activities of multiple biological processes in this developmental process.

### Common transcriptome regulation in the AR development process

**Figure 4.**
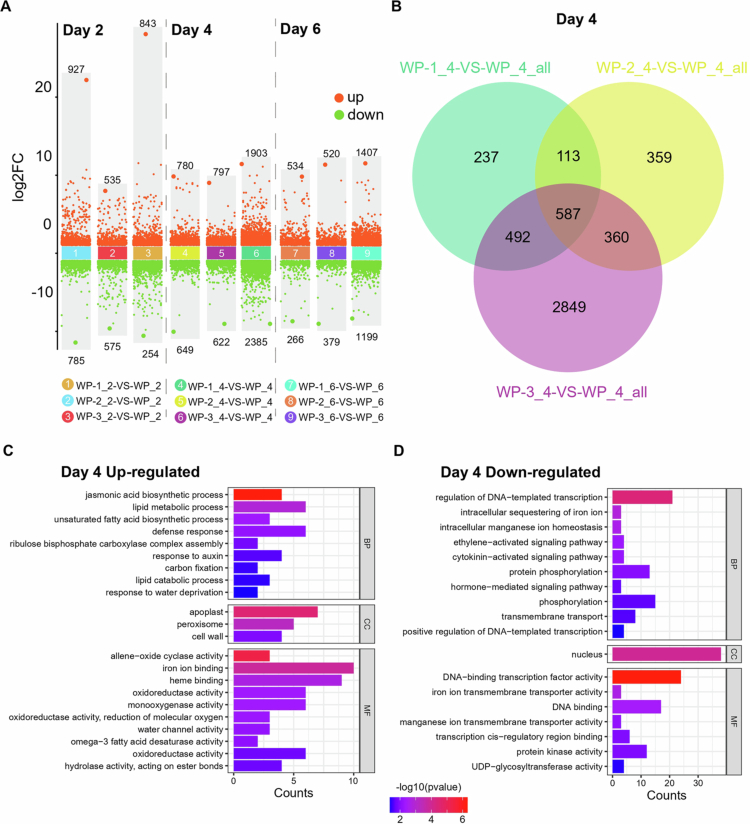
Differentially expressed genes at Day 4 were enriched with phytohormones. (A) Differential gene expression analysis showed up-regulated and down-regulated genes in all nine comparison groups on different days. (B) The Venn diagram displays the genes expressed in three different comparison groups on Day 4. (C) A total of 233 genes commonly up-regulated in WP-1, WP-2, and WP-3 subjected to Enrichment of GO. (D) A total of 139 genes commonly down-regulated in WP-1, WP-2, and WP-3 subjected to Enrichment of GO.

When comparing WP-1, WP-2, and WP-3 with WP on the second day, the three treatment materials had 1712, 1110, and 1097 DEGs, respectively. On the fourth day, there were 1429, 1419, and 4288 DEGs in the three groups, making it the day with the highest number of DEGs out of the three days. On the sixth day, the numbers of DEGs were 800, 899, and 2606, respectively (Figure 4A).

The highest number of DEGs was found on the fourth day, and the Venn diagram results show that there were 587 common genes shared by the three groups (Figure 4B). Among these, 233 genes were significantly upregulated, and the GO enrichment analysis revealed that they were related to the JA biosynthetic process and response to auxin. The genes involved in the biochemical synthesis of JA include JA biosynthetic enzyme AOC (CmoCh04G029760, CmoCh15G010360, and CmoCh15G002030) and 12-OXOPHYTODIENOATE REDUCTASE 3-like (OPRIII) (CmoCh10G007910). The transcription factors that respond to auxin include MYB306 (CmoCh01G008230, CmoCh11G001610), SAUR24 (CmoCh20G008010), and SAUR71 (CmoCh20G002990) (Figure S2). Similarly, 139 genes that were significantly downregulated were enriched in pathways such as the ethylene-activated signaling pathway, cytokinin-activated signaling pathway, and hormone-mediated signaling pathway (Figure 4C,D). The commonly downregulated genes included the ethylene response factors ERF5 (CmoCh06G008250, CmoCh16G011680) and ERF105 (CmoCh15G014220, CmoCh02G009910), zinc finger proteins related to the cytokinin-activated signaling pathway (CmoCh18G010170, CmoCh04G016710), and signal regulatory factor ARR5 (CmoCh04G016750, CmoCh15G010550), as well as genes involved in hormone-mediated signaling pathways (CmoCh04G002030, CmoCh17G001200, CmoCh07G007890) (Figure S2, Data S2).

On the second day, 30 common upregulated genes and 68 common downregulated genes were significantly enriched in the regulation of DNA-templated transcription, proteolysis, cell differentiation, and defense response (Figure S3A,B). The GO analysis results on the sixth day showed that 85 commonly upregulated genes were significantly enriched in the cytokinin-activated signaling pathway (CmoCh15G008650, CmoCh04G022980, and CmoCh11G002390), while 26 commonly downregulated genes were enriched in the ethylene-activated signaling pathway (CmoCh08G002930 and CmoCh07G002060) (Figures S2 and S3C,D, Data S2).

Transcription factors regulate the formation and development of ARs through various mechanisms, directly or indirectly, including influencing the direction of cell division, auxin signal transduction, and interactions with other plant hormones.^12^^,^^38^ We counted the transcription factors that were significantly expressed during the development of ARs over these three days. On the second day, NAC was the most upregulated transcription factor, followed by WRKY and Dof (Figure S4A), while MYB, NAC, and GATA were ranked as the top three most downregulated transcription factors (Figure S4B). The number of transcription factors on the fourth day was the highest among the three days, with bHLH, ERF, MYB, and NAC being the most prominent (Figure S4C,D). Similarly, ERF, bHLH, and NAC accounted for the majority of the transcription factors on the sixth day (Figure S4E,F). In summary, the expression changes in these transcription factors at different time points reflect the dynamic regulatory network during AR formation, revealing their key roles at different stages.

### Activated phytohormone signaling pathways during AR formation

**Figure 5.**
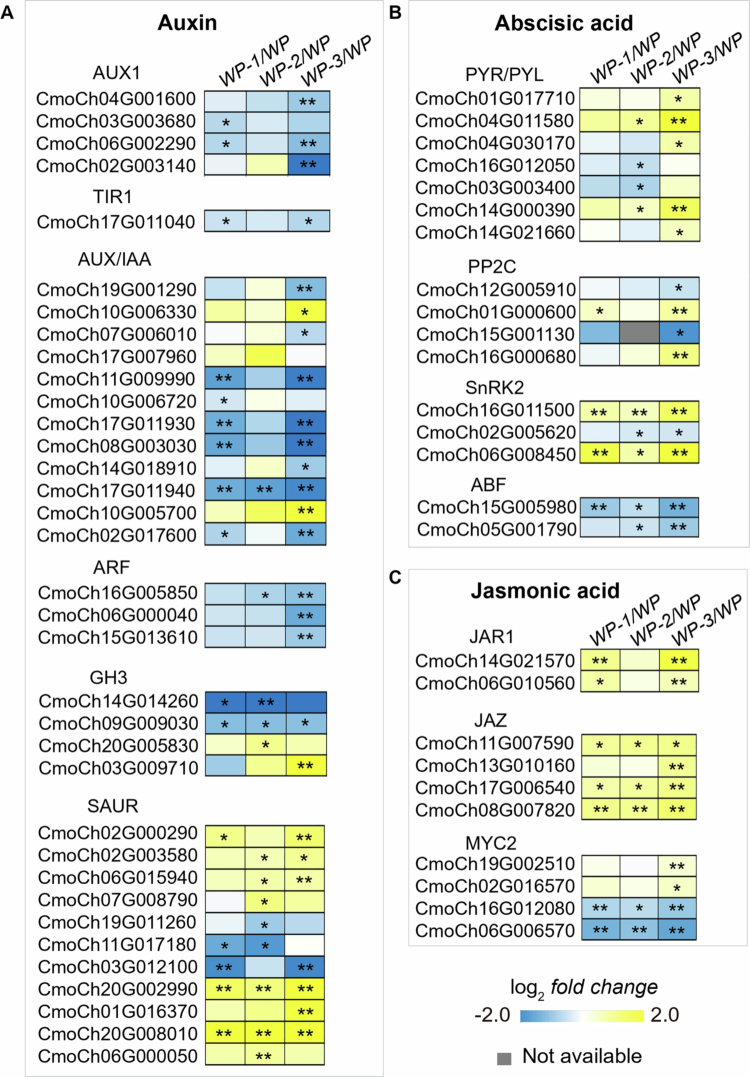
Expression of key genes in phytohormone signaling pathways. Heatmaps show log-transformed fold changes in the expression of DEGs in the auxin (A), abscisic acid (B), jasmonic acid (C) signaling pathways in three different comparison groups on Day 4. The blue–yellow color gradient represents the log2FC values between WP-1, WP-2, WP-3, and WP. * indicates *p* < 0.05; ** indicates *p* < 0.01. Gray blocks mean no data were available.

Both the GO enrichment analysis of common differential genes and the expression distribution of transcription factors indicate that plant hormones play an important role in the formation of ARs. We thus identified the expression levels of DEGs related to key components of the plant hormone signaling pathway (ko04075), identifying a total of 82 common hormone-related DEGs, including five pathways involving auxin, ABA, JA, cytokinins, and ethylene. The DEGs related to the auxin pathway accounted for the largest proportion, totaling 35. Compared to WP, most genes related to auxin transporter AUX1, auxin receptor TIR1 protein, transcriptional repressor Aux/IAA protein, transcription factor ARF, and genes encoding auxin amide synthetase GH3 were downregulated in WP-1, WP-2, and WP-3. *SAUR*, as a late response gene, may be involved in transcription and post-translational protein expression. Except for a few genes, most involved in SAUR were significantly upregulated (Figure 5A). In the ABA signaling pathway, 16 annotated genes showed differential expression: PYR/PYL receptor genes were significantly upregulated in WP-3, and SnRK2 kinase genes were highly expressed, while PP2C phosphatases and ABF transcription factors were mostly downregulated across the three treatment groups (Figure 5B). JA synthase JAR1 and transcriptional repressor JAZ were significantly upregulated, while MYC2, as a transcriptional activator, showed both upregulation and downregulation (Figure 5C).

### Key modules and candidate genes associated with AR formation

**Figure 6.**
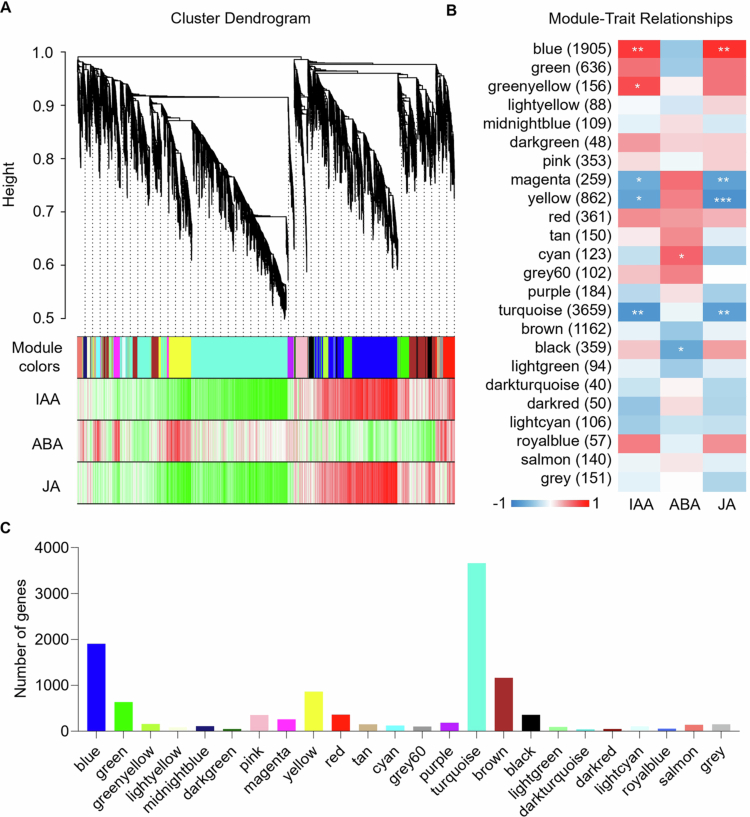
Identifying modules related to phytohormones with WGCNA. (A) Weighted gene co-expression network analysis identified a hierarchical cluster tree showing co-expression modules, while the major tree branches constituted 24 modules labelled using different colors. (B) Module‒trait relationship diagram. The left panel displays the genes matched by 24 modules. The blue‒red color gradient represents the module feature correlation. Asterisks indicate the correlation between module samples and the corresponding *p*-value. * indicates *p* < 0.05; ** indicates *p* < 0.01. (C) The number of genes in each module.

To further explore the relationship between hormone levels and the regulatory network of candidate genes during AR development, we performed WGCNA.^39^ The genes with similar expression patterns were divided into 24 modules, each assigned a different color (Figure 6A). There were significant differences in the number of DEGs across different modules. The turquoise module contained the most DEGs, totaling 3659, followed by the blue (1905) and brown modules (1162). In contrast, the darkturquoise, darkgreen, and darkred modules had the lowest DEG counts, with 40, 48, and 50, respectively (Figure 6C). The module‒trait relationship results showed that seven identified modules were significantly correlated with the content of three hormones. The blue module showed significant positive correlations with IAA and JA; the greenyellow module with IAA; and the cyan module with ABA (Figure 6B).

**Figure 7.**
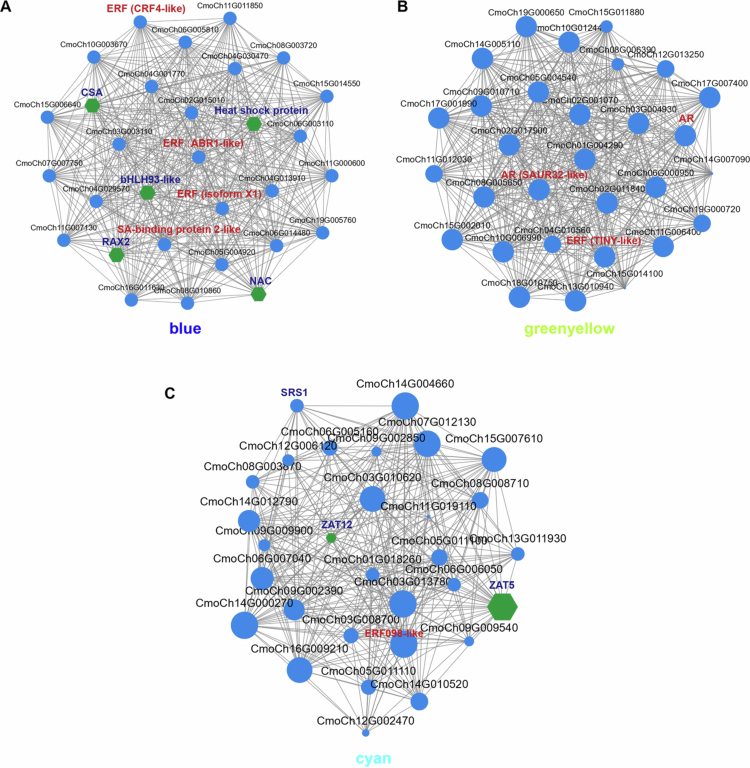
Screening of the co-expression network of central genes. Hub co-expression gene network for the blue (A), greenyellow (B), and cyan modules (C). Each dot represents a gene, each line represents the regulatory relationship between them. Genes related to phytohormones and transcription factors are represented in bold font of different colours and shapes.

Based on the three central modules mentioned above, we constructed a DEG co-expression network by selecting each module's top 30 most correlated genes to identify key regulatory genes. By using Cytoscape to create and visualize the gene network,^39^ the key regulatory genes in the blue, greenyellow, and cyan modules were identified. In this network, each node represents a gene, and the edges connecting them indicate their co-expression correlation. The central genes with the closest connections in this network may be key regulatory genes, selected based on high weights and values from the core genes of the modules (Data S3). In the blue module, the ethylene-related transcription factors ERF (CRF4 and ABR1) and salicylic acid-binding protein 2 were identified, along with the transcription factors CSA, HSP, bHLH93, and RAX2 (Figure 7A). The auxin response factor SAUR32 and the ethylene transcription factor TINY were identified in the greenyellow module (Figure 7B). In the cyan module, which is positively correlated with ABA, the ethylene transcription factor ERF098 and the zinc finger binding proteins ZAT5 and ZAT12 were identified (Figure 7C).

We also performed WGCNA on the data from the second and sixth days. The tan and darkgrey modules were the ones with the highest and most significant correlations with ABA and JA on the second day, respectively (Figure S5A,B). In the tan module, the transcription factors C3H and MYB52 were identified (Figure S5C), while in the darkgrey module, WRKY and the ethylene transcription factor ERF054 (Figure S5D) were identified. The results from the sixth day showed that the salmon module was significantly positively correlated with ABA, and the brown module was significantly positively correlated with JA (Figure S6A,B). The auxin response factor SAUR21, the transcription factor MYB, and G2-like were identified in the candidate gene regulatory network of the salmon module (Figure S6C). In the brown module, the auxin response factor SAUR32, the ethylene transcription factor ABR1, and the transcription factor NAC were identified (Figure S6D).

### Confirmation of transcriptome data through qRT-PCR

To verify the accuracy of the transcriptome data, we conducted validation using quantitative real-time PCR. From numerous DEGs, we selected eight key genes as objects for validation. These included hormone-related genes, ERFs (CmoCh08G002930 and CmoCh02G017160), jasmonate (CmoCh01G019880 and CmoCh08G007820), the hormone-mediated signaling pathway (CmoCh04G002030), and salicylic acid (CmoCh18G001780), as well as the key transcription factor bHLH (CmoCh06G006570) and mitogen-activated protein kinase (CmoCh01G005840) (Figure 8A–H). The qRT‒PCR results verified the transcriptomic data, showing consistent expression trends for the selected target genes across materials despite quantitative variations.

## Discussion

**Figure 8.**
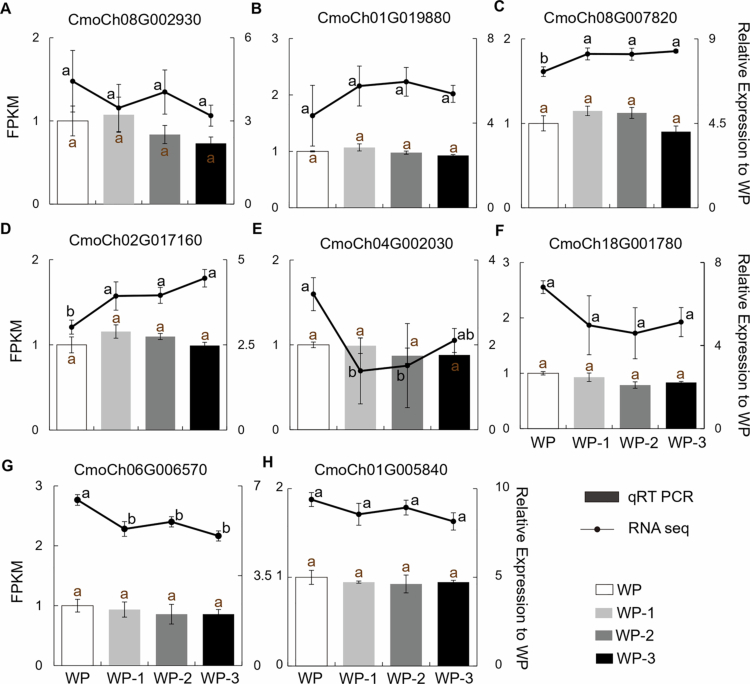
qRT‒PCR validation of RNA-seq data. Quantitative real-time PCR (qRT‒PCR) was conducted to assess the transcript levels of selected phytohormone genes (A‒H) (CmoCh08G002930, CmoCh01G019880, CmoCh08G007820, CmoCh02G017160, CmoCh04G002030, and CmoCh18G001780), transcription factors (CmoCh06G006570) and mitogen-activated protein kinase (CmoCh01G005840) in WP WP-1, WP-2, and WP-3 at Day 4. Error bars represent standard deviations of three biological replicates. Different letters within different days indicate statistically significant differences based on one-way ANOVA.

### Discussion Unveiling the role of scions in the coordinated regulation of ar development by phytohormones

Pumpkin is the preferred watermelon rootstock due to its efficient root system, which enhances yield, fruit quality, and grafting success through strong compatibility. While rootstocks' effects on scions are well-documented, scions' influence on rootstocks is less studied, with limited research on crops like apple and grape.^40^^,^^41^ Our study investigated how the “HXX” watermelon scion affects AR development in pumpkin rootstock hypocotyls. We found that removing the scion’s cotyledons significantly boosted the AR number, length, and biomass (Figure 1), suggesting that cotyledons inhibit AR formation, possibly via hormone signals or resource competition. This finding is particularly novel as the influence of scions on rootstock roots is less studied, and it contrasts with research in tomato, in which it has been reported that scion leaf removal inhibited adventitious rooting on the scion itself, highlighting the species- and location-specific nature of this regulation.^42^ The inhibitory effect observed in our system may be due to the cotyledons acting as a source of suppressive signals or as a competitive resource sink. The role of cotyledons as a key hormonal signaling center has been established in Arabidopsis, where their removal altered auxin levels and affected graft union formation.^43^ From a resource allocation perspective, scion cotyledons are a strong energy sink; their removal may redirect resources to the rootstock to fuel AR development. This concept is supported by studies where removing rootstock cotyledons limited graft success due to insufficient energy reserves.^44^ Our study revealed that manipulating these scion organs significantly alters the levels of key hormones (IAA, ABA, and JA) in the rootstock, which are critical for AR formation (Figure 2). This interaction likely arises from dynamic shifts in hormone synthesis and resource allocation between the scion and rootstock. The observed hormonal changes, such as elevated IAA and JA in the rootstock, may promote cell division and stress resilience, thereby facilitating AR initiation and growth. Fluctuations in ABA levels suggest that cotyledons could be a significant ABA source, while JA dynamics might balance growth promotion with defense needs during AR development.

The hormone discrepancy between the rootstock and scion arises from the differential regulation of hormone metabolism and signaling during grafting and scion manipulation (Figure S1). The differing trends in hormone ratios between the WP-1 rootstock and scion likely stem from distinct metabolic activities in each tissue. Cotyledon removal in WP-1 might trigger rootstock-specific metabolic pathways favoring IAA accumulation relative to ABA and JA, promoting rooting, which is not mirrored in the scion. While scion-derived signals, including hormones, are known to affect rootstock activity,^7^ our results align with recent findings showing that the scion's overall hormone balance does not directly dictate the rootstock's ratios, as seen with ABA in grapevine.^8^ This is because the rootstock can also synthesize its own hormones and respond uniquely to grafting signals, possessing its own complex gene regulatory networks for development.^45^ Furthermore, our bulk tissue measurements may mask dynamic hormone changes at AR initiation sites, where localized hormone maxima critically regulate cell fate determination and primordium formation.^46^ The divergent hormonal responses from the scion and rootstock seen in WP-1 reflect differential stress adaptation and resource allocation; grafting-induced stress uniquely mobilizes physiological pathways in each tissue.^47^ Ultimately, the observed differences demonstrate the complex physiological interplay between the scion and rootstock in response to grafting and scion manipulations. This interconnected system regulates the hormone balance to control AR development. However, these phenotypic findings require molecular studies to uncover scion-rootstock root regulation mechanisms.

Auxins are well-established drivers of cell division and root primordium formation in AR development.^48^ Our transcriptome analysis supports this fundamental role, highlighting enriched auxin-activated signaling pathways (Figure 3C), which reinforces auxin's pivotal role in AR initiation. However, AR regulation is complex and extends beyond auxin, involving intricate interactions with other hormones. Our findings suggest that other hormones fine-tune this process, balancing growth and stress responses. For instance, the WP-1 treatment, which resulted in improved AR formation, correlated with dynamic changes in JA levels (Figure 2C) and the significant upregulation of JA-related genes (Figure 5C). This aligns with previous studies indicating that JA can enhance auxin-induced AR formation, particularly under stress conditions such as wounding.^46^^,^^49^^,^^50^ JA has been shown to upregulate genes involved in auxin biosynthesis, thereby promoting AR development in petunia cuttings and tobacco thin cell layers, especially under stress.^46^^,^^50^ Given that grafting involves wounding, which activates JA signaling, it is plausible that JA contributes to AR formation in our system by modulating auxin pathways. However, as these findings are derived from different plant species, further investigation is needed to confirm JA’s specific role in watermelon grafting. Furthermore, our data indicate the significant downregulation of common genes enriched in cytokinin- and ethylene-related pathways (Figure 4D), implying their potential involvement in a coordinated hormonal network that modulates auxin-mediated AR development. Contextualized with prior research, these findings reveal a dynamic regulatory framework: auxin drives AR development in hypocotyls, which is modulated through crosstalk with JA, cytokinins, and ethylene.

### The effect of transcription factors on AR development during scion-rootstock interaction

Scion‒rootstock interactions regulate AR formation by modulating transcription factors through scion-derived hormonal signals in grafted watermelon seedlings. The mobile transcription factor CmHY5 was shown to transfer from a melon scion to a squash rootstock, directly regulating nitrate uptake by interacting with rootstock transcription factors.^51^ Zhang et al. also showed that light conditions affect scion‒rootstock communication in cucumber, implying transcription factor involvement in root regeneration under the influence of scions.^5^ These findings reveal a complex signaling network in which scion-derived hormones regulate rootstock transcription factor expression, driving AR formation (Figure S4).

Transcriptome sequencing revealed a suite of TFs implicated in AR formation (Figure S4), consistent with their established roles in root differentiation across plant species.^48^ Our analysis identified TFs from the ARF, AP2/ERF, MYB, NAC, WRKY, GRAS, and bHLH families, with bHLH being the most prevalent. This prominence aligns with studies in Arabidopsis, where bHLH members PFA and PFB orchestrate pericycle cell competence for lateral root initiation. PFA overexpression triggers auxin-induced cell division and pericycle-specific gene expression, whereas PFB suppression impairs these processes, highlighting bHLH's regulatory influence on root primordium development.^52^ Similarly, AP2/ERF, the second most abundant in our dataset, are critical in AR regulation. WGCNA pinpointed ERFs such as ERF054 (Figure S5), ERF098 (Figure 7), and others (Figure S6), which are known to integrate stress and hormonal cues. For instance, *WOX* genes amplify ERF054 and ERF034 expression, enhancing root formation.^53^ In chestnut, ERF098 is active during the early AR and callus phases, yet in mature buds, ERF098 and ERF107 may suppress ARs by modulating JA or cytokinin signals, suggesting a role in hormone crosstalk, particularly with ethylene.^54^ NAC also plays a significant role, mirroring their involvement in Arabidopsis leaf explant-derived ARs.^55^ Research shows that *NAC* genes drive cell elongation in rose petals and roots.^56^ However, *NAC1* is induced alongside auxin pathways to promote root tip emergence in wounded Arabidopsis.^57^ This parallels our findings, indicating NAC’s contribution to AR initiation in grafted seedlings. Meanwhile, MYB, expressed across AR development stages, exhibits structural and functional diversity. Though their root-specific roles are less defined, studies like that investigating PuMYB40 in *Populus ussuriensis* demonstrate MYB’s capacity to enhance AR formation under phosphorus starvation.^58^ Our findings suggest that bHLH, AP2/ERF, NAC, and MYB collectively mediate hormone signaling and stress responses to shape AR outcomes. Future investigations should explore TF expression dynamics during grafting and their interplay with other regulatory elements. This will establish a foundation for enhancing grafting success and guiding plant breeding strategies.

This study reveals that targeted scion cotyledon removal specifically promotes AR development in grafted watermelon rootstocks. This effect is achieved by inducing favorable shifts in rootstock hormone levels, specifically those of IAA, ABA, and JA, coupled with significant transcriptomic reprogramming, which activates key TF families within the rootstock, including bHLH, ERF, MYB, NAC, and SAURs. These findings underscore the scion's active regulatory role, and particularly the inhibitory effect of its cotyledons in this system, and offer molecular insights for potentially optimizing grafting techniques. However, the exact interactions between these hormones following specific scion manipulations and their precise role in regulating AR development remain insufficiently explored. Furthermore, these analyses were performed on bulk scion and rootstock tissues, which may have obscured any highly localized molecular and hormonal fluctuations essential for AR initiation at specific cellular sites. Our transcriptome profiling commenced at the developmental onset of ARs postgrafting to capture the dynamic changes during AR development influenced by the scion rather than the immediate response to grafting wounds. Consequently, while our findings establish strong correlations and provide initial evidence for the scion's regulatory role, future studies are needed to refine our understanding of these intricate mechanisms.

## Conclusion

This study provides preliminary insights into the significant role of scion cotyledons in regulating adventitious root development in grafted watermelon seedlings. Cotyledon removal significantly boosted AR formation, suggesting that they have an inhibitory role, while true leaves have a minor impact. The scion leaf treatments altered the rootstock hormone levels, implicating IAA, ABA, and JA in AR development. Transcriptome analysis identified key transcription factors as potential targets for regulating adventitious rooting. Further research is needed to fully understand these mechanisms. Practically, our work indicates that early-stage management of scion cotyledons should be considered in watermelon graft cultivation to enhance root system robustness. These preliminary understandings establish a molecular framework for the complex interplay among scion leaves, hormonal signaling, and transcriptional regulation in grafting. This foundation enables future optimization of techniques to improve seedling survival, early vigor, and overall efficiency in watermelon production.

## Supplementary Material

Supplementary material Hub genes list of salmon and brown module by WGCNA correlated with measured endogenous hormone at Day 6. Selected 30 genes from each module.

Supplementary material Commonly down-regulated genes in WP-1, WP-2 and WP-3. The list shows a total of 26 genes that were commonly downregulated (Log2FC<-1, P<0.05) in WP-1, WP-2, and WP-3 at Day 6. Relative level of transcript compared with WP were colored according to Log2FC values: -2 (blue) to 2 (yellow).

Supplementary material Data S1 Transcript level of 32,462 genes in WP, WP-1, WP-2 and WP-3 by RNA deep sequencing.

Supplementary material Figure S1. Changes in endogenous hormone content in scion at different stages. (A–C) Changes in endogenous content of IAA, ABA, and JA in the four phenotypes on Days 2, 4, and 6. (D and E) Temporal dynamics of phytohormone ratios IAA to ABA and JA are shown from Day 0 to Day 6 post-grafting. Error bars indicate the standard deviation across three biological replicates. Different letters within different days indicate statistically significant differences based on one-way ANOVA. The dots represent biological replicates.

Supplementary material Figure S2. Transcript level of phytohormone genes analyzed by GO enrichment. Log-transformed fold changes in transcript levels for genes related to phytohormones by GO enrichment in WP-1, WP-2, and WP-3 at Day 4 and Day 6 (* indicates *p* < 0.05, and ** indicates *p* < 0.01).

Supplementary material Figure S3. Commonly up-regulated and down-regulated genes in WP-1, WP-2, and WP-3 are enriched by GO analysis at Day 2 and Day 6. (A and B) A total of 30 genes commonly up-regulated and 68 genes down-regulated in WP-1, WP-2, and WP-3 were subjected to Enrichment of GO at Day 2. (C and D) A total of 85 genes commonly up-regulated and 26 genes down-regulated in WP-1, WP-2, and WP-3 were subjected to Enrichment of GO.

Supplementary material Figure S4. Number of transcription factors in commonly expression of DEGs on different days. (A and B) The number of transcription factors in DEGs that were commonly and significantly up-regulated or down-regulated in WP-1, WP-2, and WP-3 at Day 2. (C and D) The number of transcription factors in DEGs that were commonly and significantly up-regulated or down-regulated in WP-1, WP-2, and WP-3 at Day 4. (E and F) The number of transcription factors in DEGs that were commonly and significantly up-regulated or down-regulated in WP-1, WP-2, and WP-3 at Day 6.

Supplementary material Figure S5. Identifying modules related to phytohormones and screening of co-expression network of central genes at Day 2 with WGCNA. (A) WGCNA identified a hierarchical cluster tree showing co-expression modules, while the major tree branches constituted 25 modules labeled using different colors. (B) Module‒trait relationship diagram. The left panel displays the genes matched by 25 modules. The blue‒red color gradient represents the module feature correlation. Asterisks indicate the correlation between module samples and the corresponding *p*-value. * indicates *p* < 0.05; ** indicates *p* < 0.01. (C and D) Hub co-expression gene network for the tan and darkgrey module at Day 2. Each dot represents a gene, and each line represents the regulatory relationship between them. Genes related to phytohormones and transcription factors are represented in bold font of different colors and shapes.

Supplementary material Figure S6. Identifying modules related to phytohormones and screening of co-expression network of central genes at Day 6 with WGCNA. (A) WGCNA identified a hierarchical cluster tree showing co-expression modules, while the major tree branches constituted 20 modules labeled using different colors. (B) Module‒trait relationship diagram. The left panel displays the genes matched by 20 modules. The blue‒red color gradient represents the module feature correlation. Asterisks indicate the correlation between module samples and the corresponding *p*-value. * indicates *p* < 0.05; ** indicates *p* < 0.01. (C and D) Hub co-expression gene network for the salmon and brown module at Day 6. Each dot represents a gene, and each line represents the regulatory relationship between them. Genes related to phytohormones and transcription factors are represented in bold font of different colors and shapes.

Supplementary material Table S1. List of Preprocessing Results for Sequencing Data Quality.Table S2. Statistical results of the alignment rate between reads and reference sequence. Table S3. Primers used for qRT‒PCR in this study.

## Open accessible data

RNA-seq data PRJNA1202060 accessed at https://dataview.ncbi.nlm.nih.gov/object/PRJNA1202060?reviewer = nrhqrklqql1dc7kr6bhq1jip60.
