# Supplementary figures and images for: Scion organ removal alters hormone levels and gene expression associated with adventitious root development in grafted watermelon seedlings
Source: Plant Signal Behav. 2025 Sep 12;20(1):2556300. doi: 10.1080/15592324.2025.2556300 (PMC12439556; doi:10.1080/15592324.2025.2556300)

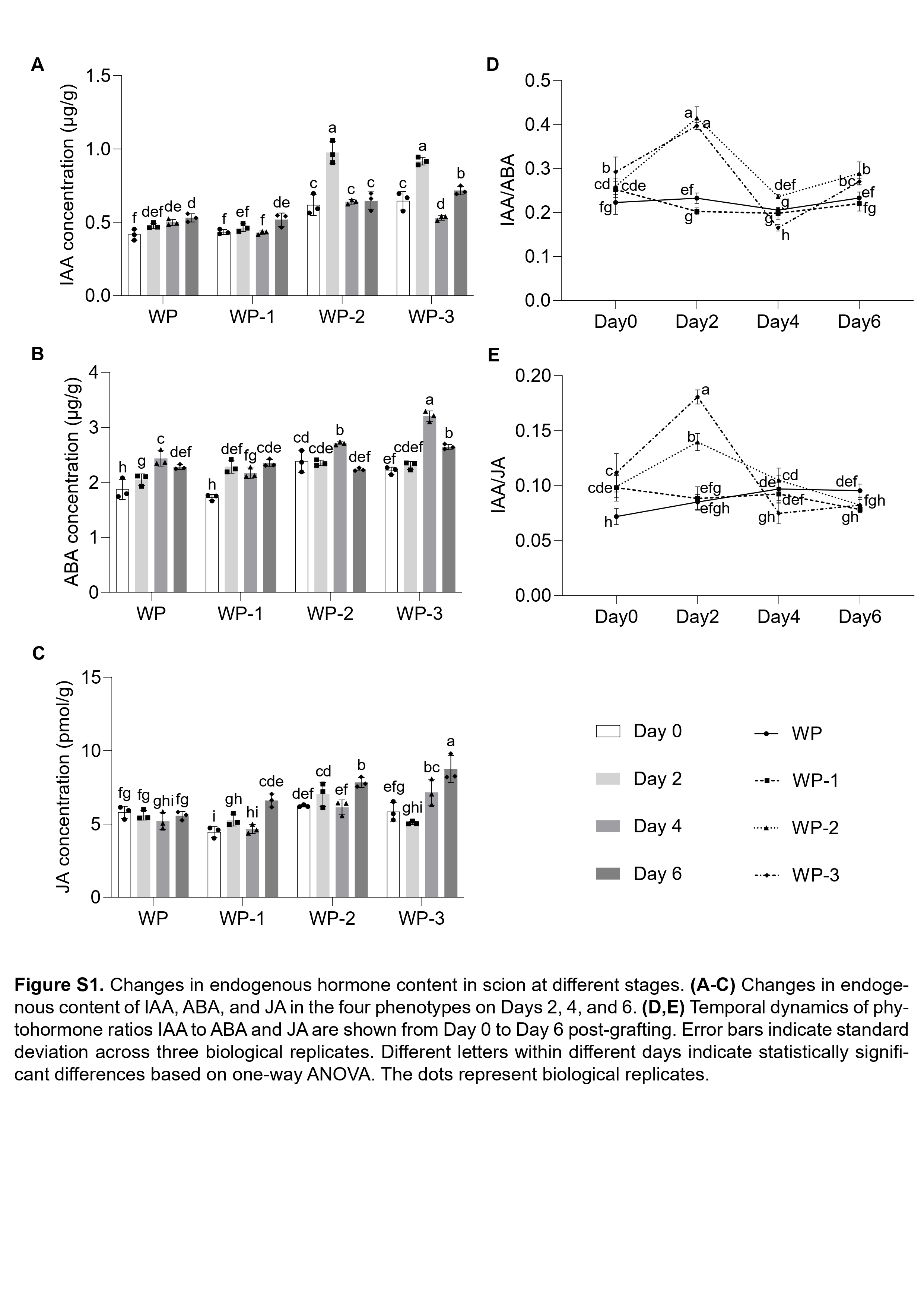

Supplement: Supplementary material — Figure S1. Changes in endogenous hormone content in scion at different stages. (A–C) Changes in endogenous content of IAA, ABA, and JA in the four phenotypes on Days 2, 4, and 6. (D and E) Temporal dynamics of phytohormone ratios IAA to ABA and JA are shown from Day 0 to Day 6 post-grafting. Error bars indicate the standard deviation across three biological replicates. Different letters within different days indicate statistically significant differences based on one-way ANOVA. The dots represent biological replicates. [file KPSB_A_2556300_SM0875.jpg]

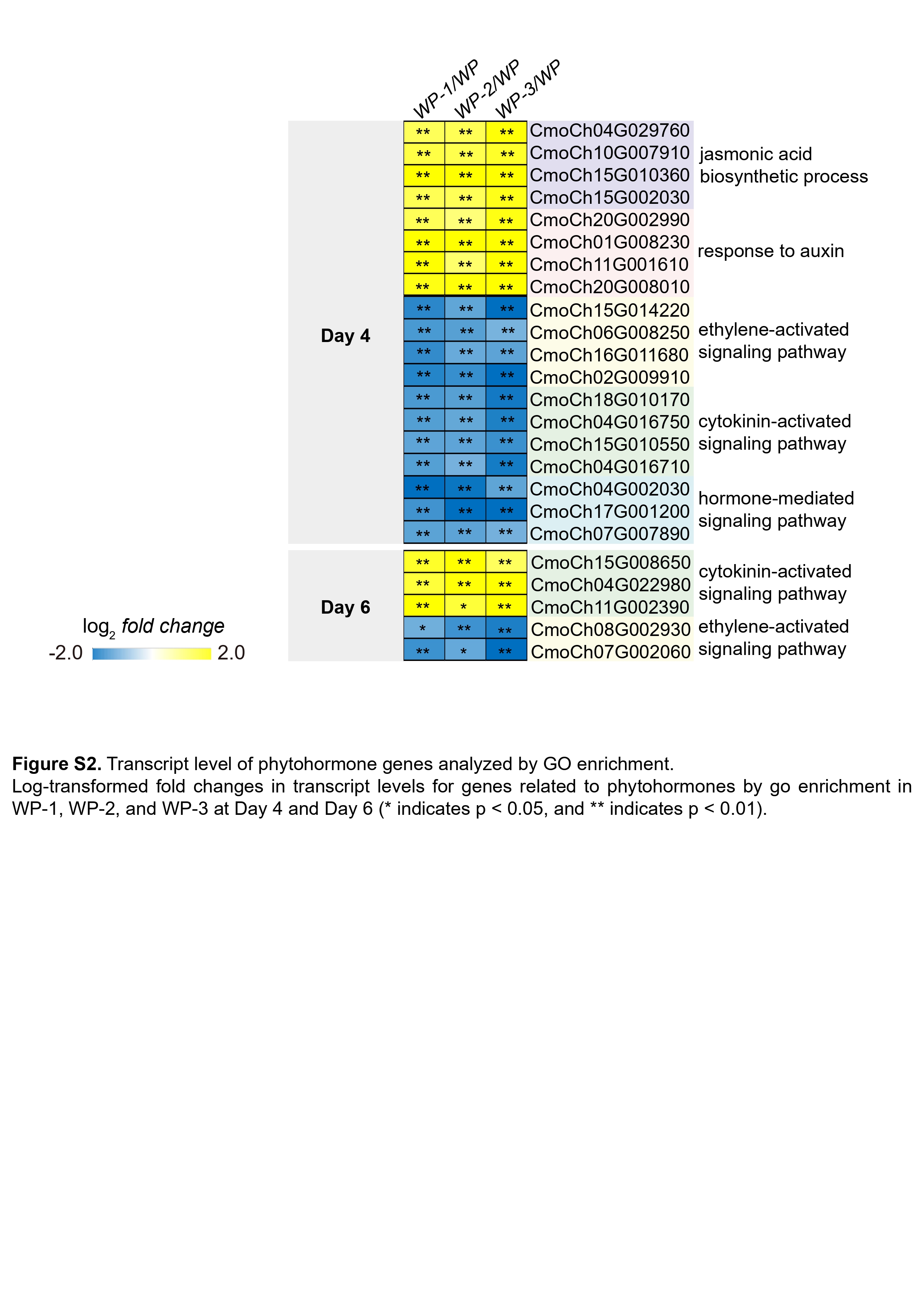

Supplement: Supplementary material — Figure S2. Transcript level of phytohormone genes analyzed by GO enrichment. Log-transformed fold changes in transcript levels for genes related to phytohormones by GO enrichment in WP-1, WP-2, and WP-3 at Day 4 and Day 6 (* indicates p < 0.05, and ** indicates p < 0.01). [file KPSB_A_2556300_SM0876.jpg]

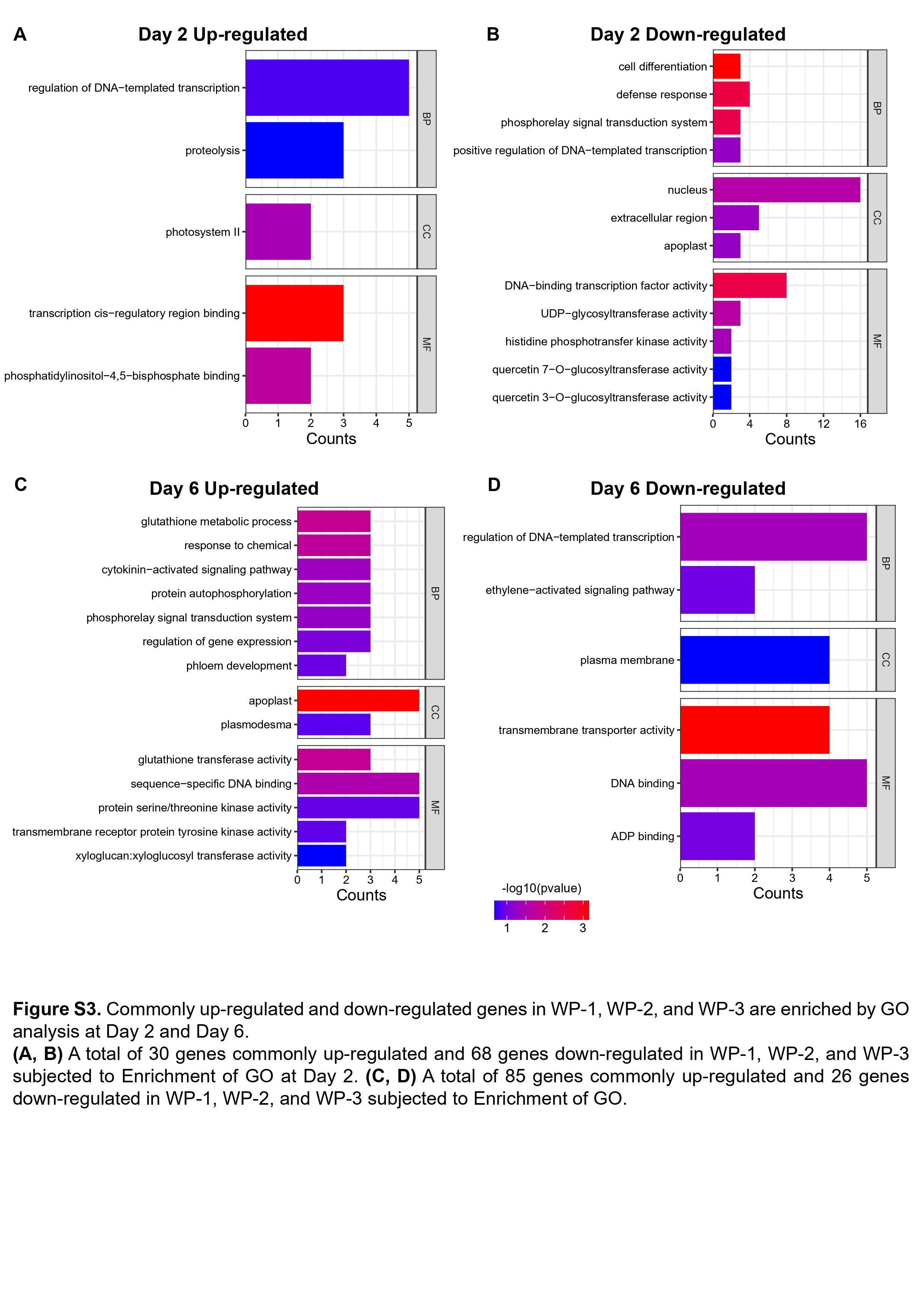

Supplement: Supplementary material — Figure S3. Commonly up-regulated and down-regulated genes in WP-1, WP-2, and WP-3 are enriched by GO analysis at Day 2 and Day 6. (A and B) A total of 30 genes commonly up-regulated and 68 genes down-regulated in WP-1, WP-2, and WP-3 were subjected to Enrichment of GO at Day 2. (C and D) A total of 85 genes commonly up-regulated and 26 genes down-regulated in WP-1, WP-2, and WP-3 were subjected to Enrichment of GO. [file KPSB_A_2556300_SM0882.jpg]

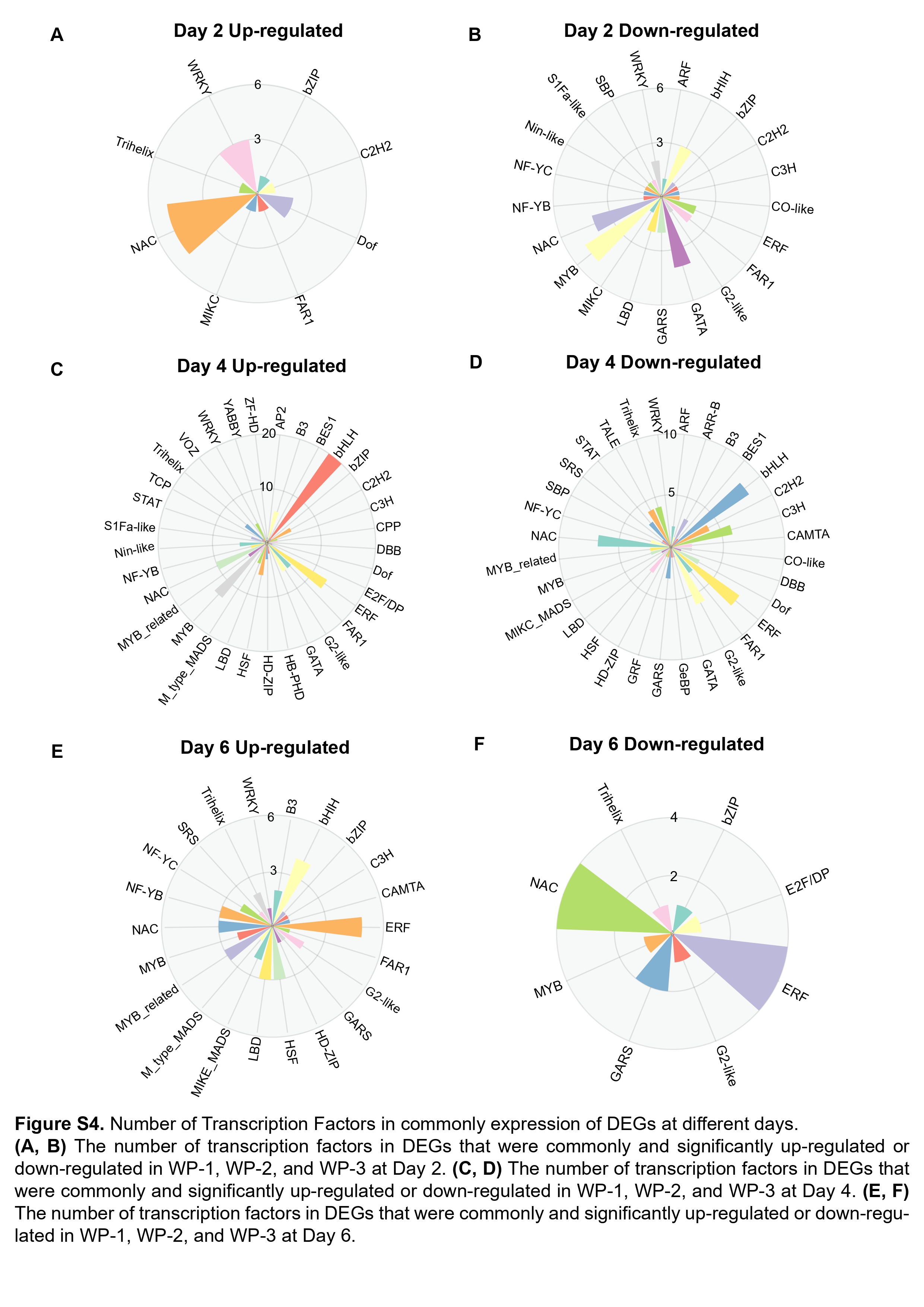

Supplement: Supplementary material — Figure S4. Number of transcription factors in commonly expression of DEGs on different days. (A and B) The number of transcription factors in DEGs that were commonly and significantly up-regulated or down-regulated in WP-1, WP-2, and WP-3 at Day 2. (C and D) The number of transcription factors in DEGs that were commonly and significantly up-regulated or down-regulated in WP-1, WP-2, and WP-3 at Day 4. (E and F) The number of transcription factors in DEGs that were commonly and significantly up-regulated or down-regulated in WP-1, WP-2, and WP-3 at Day 6. [file KPSB_A_2556300_SM0886.jpg]

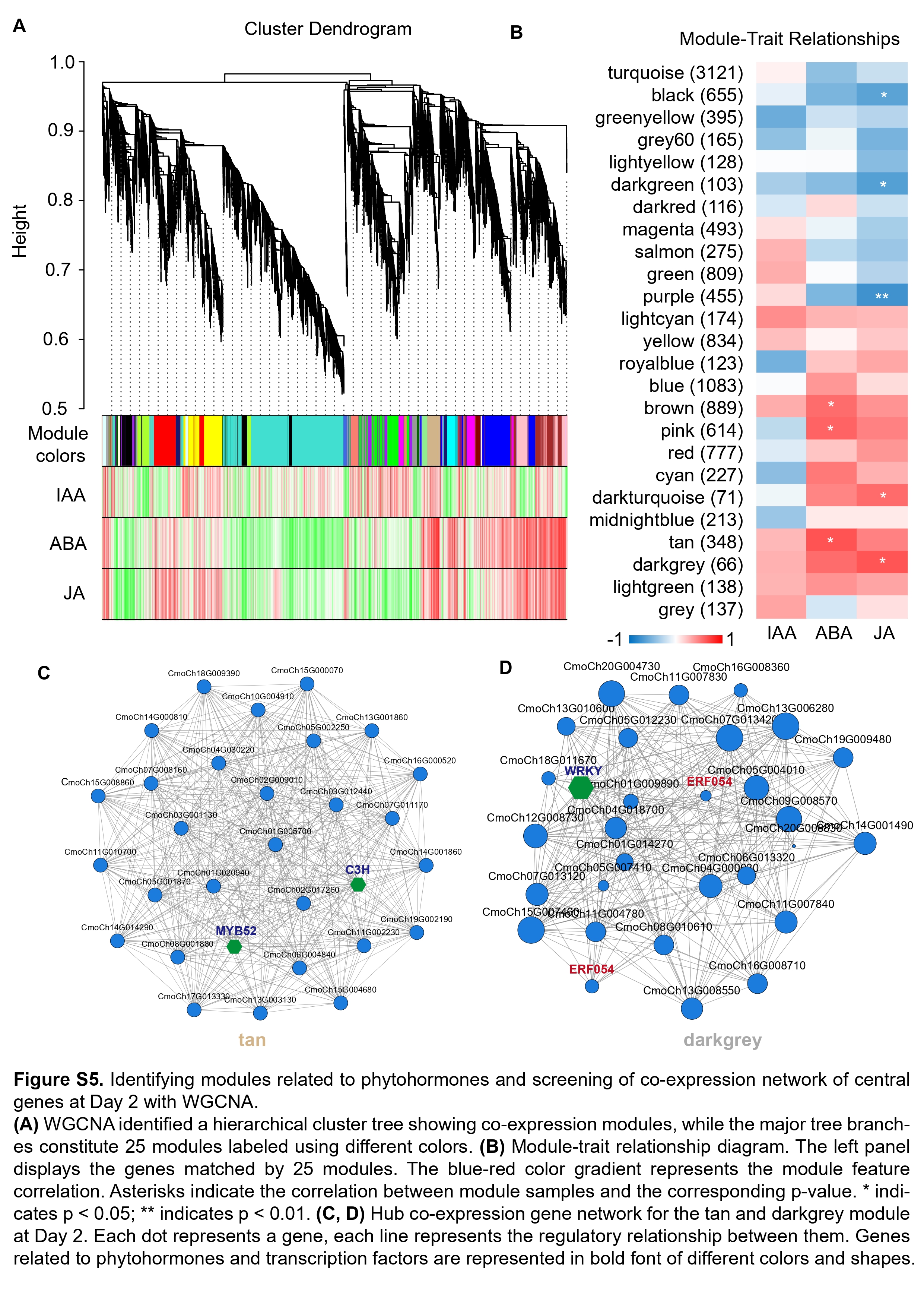

Supplement: Supplementary material — Figure S5. Identifying modules related to phytohormones and screening of co-expression network of central genes at Day 2 with WGCNA. (A) WGCNA identified a hierarchical cluster tree showing co-expression modules, while the major tree branches constituted 25 modules labeled using different colors. (B) Module‒trait relationship diagram. The left panel displays the genes matched by 25 modules. The blue‒red color gradient represents the module feature correlation. Asterisks indicate the correlation between module samples and the corresponding p-value. * indicates p < 0.05; ** indicates p < 0.01. (C and D) Hub co-expression gene network for the tan and darkgrey module at Day 2. Each dot represents a gene, and each line represents the regulatory relationship between them. Genes related to phytohormones and transcription factors are represented in bold font of different colors and shapes. [file KPSB_A_2556300_SM0889.jpg]

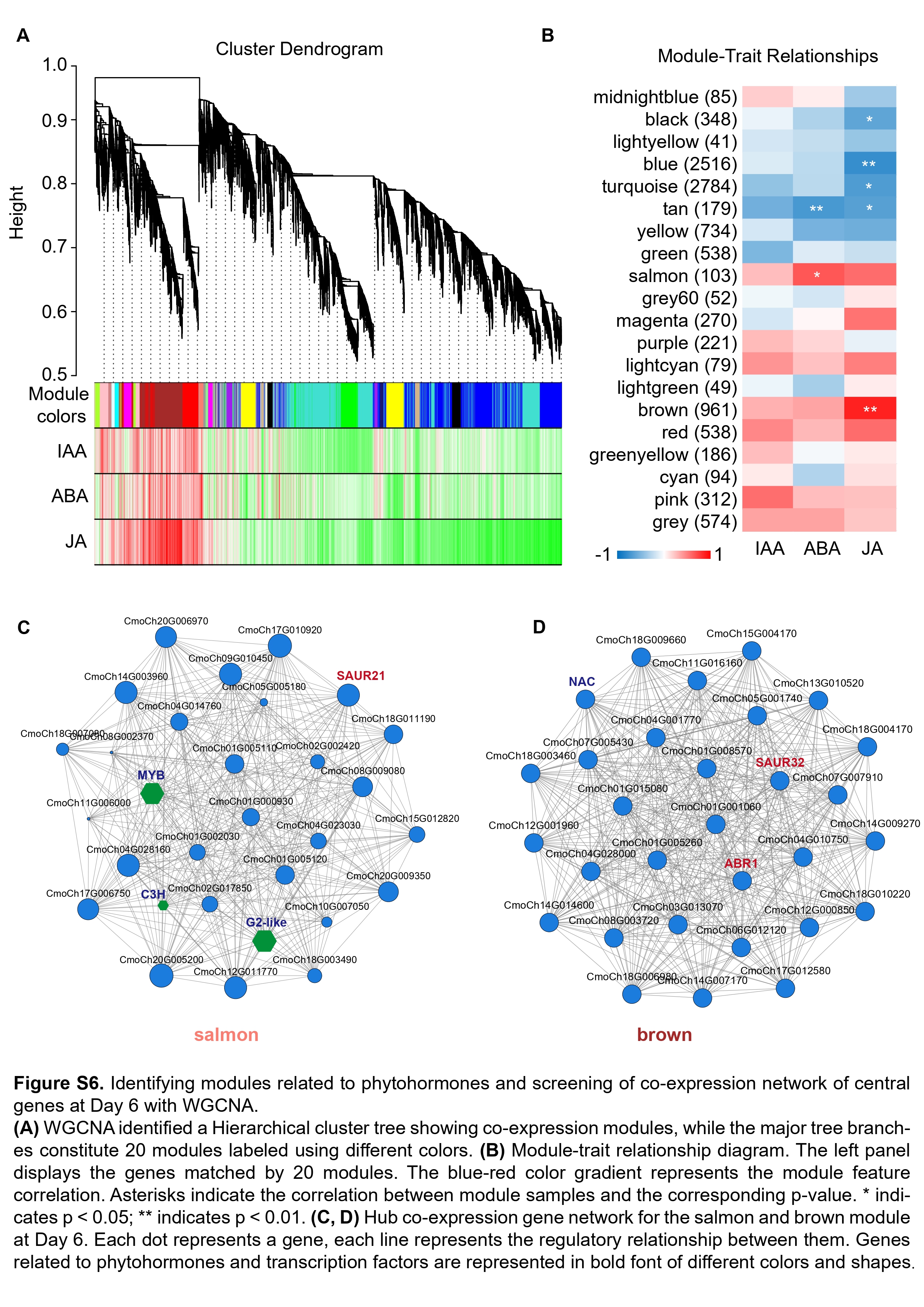

Supplement: Supplementary material — Figure S6. Identifying modules related to phytohormones and screening of co-expression network of central genes at Day 6 with WGCNA. (A) WGCNA identified a hierarchical cluster tree showing co-expression modules, while the major tree branches constituted 20 modules labeled using different colors. (B) Module‒trait relationship diagram. The left panel displays the genes matched by 20 modules. The blue‒red color gradient represents the module feature correlation. Asterisks indicate the correlation between module samples and the corresponding p-value. * indicates p < 0.05; ** indicates p < 0.01. (C and D) Hub co-expression gene network for the salmon and brown module at Day 6. Each dot represents a gene, and each line represents the regulatory relationship between them. Genes related to phytohormones and transcription factors are represented in bold font of different colors and shapes. [file KPSB_A_2556300_SM0890.jpg]
