# Supplementary material for: Scion organ removal alters hormone levels and gene expression associated with adventitious root development in grafted watermelon seedlings
Source: Plant Signal Behav. 2025 Sep 12;20(1):2556300. doi: 10.1080/15592324.2025.2556300 (PMC12439556; doi:10.1080/15592324.2025.2556300)
Supplement: Supplementary material — Table S1. List of Preprocessing Results for Sequencing Data Quality.Table S2. Statistical results of the alignment rate between reads and reference sequence. Table S3. Primers used for qRT‒PCR in this study. [file KPSB_A_2556300_SM0891.docx]

**Table S1 List of Preprocessing Results for Sequencing Data Quality**

| Samples | Total Reads | Clean Reads | Clean bases | GC Content | %>Q20 | %>Q30 |
| --- | --- | --- | --- | --- | --- | --- |
| WP_2_1 | 54,862,458 | 54,862,454 | 8,106,928,836 | 44.49% | 99.36% | 97.99% |
| WP_2_2 | 47,682,286 | 47,682,286 | 7,025,569,796 | 44.27% | 99.38% | 98.07% |
| WP_2_3 | 48,316,916 | 48,316,916 | 7,180,694,788 | 43.84% | 99.31% | 97.86% |
| WP_4_1 | 44,380,356 | 44,380,352 | 6,567,046,412 | 45.07% | 99.28% | 97.76% |
| WP_4_2 | 49,952,256 | 49,952,256 | 7,378,754,930 | 45.02% | 99.34% | 97.93% |
| WP_4_3 | 49,238,618 | 49,238,618 | 7,276,029,272 | 46.41% | 99.33% | 97.91% |
| WP_6_1 | 50,296,178 | 50,296,176 | 7,389,685,618 | 45.15% | 99.41% | 98.18% |
| WP_6_2 | 41,866,676 | 41,866,672 | 6,212,890,044 | 45.22% | 99.31% | 97.82% |
| WP_6_3 | 48,374,020 | 48,374,020 | 7,164,098,916 | 45.34% | 99.36% | 98.01% |
| WP-1_2_1 | 51,164,760 | 51,164,760 | 7,540,396,280 | 44.64% | 99.37% | 98.06% |
| WP-1_2_2 | 41,769,782 | 41,769,780 | 6,190,343,414 | 46.76% | 99.34% | 97.93% |
| WP-1_2_3 | 42,386,494 | 42,386,494 | 6,257,411,360 | 48.11% | 99.37% | 98.02% |
| WP-1_4_1 | 39,467,432 | 39,467,428 | 5,842,253,440 | 45.23% | 99.31% | 97.88% |
| WP-1_4_2 | 40,956,610 | 40,956,610 | 6,082,301,404 | 44.68% | 99.39% | 98.12% |
| WP-1_4_3 | 46,848,822 | 46,848,822 | 6,893,139,112 | 44.93% | 99.39% | 98.11% |
| WP-1_6_1 | 38,806,654 | 38,806,650 | 5,751,289,870 | 45.64% | 99.32% | 97.89% |
| WP-1_6_2 | 43,452,378 | 43,452,378 | 6,426,617,140 | 45.32% | 99.35% | 98.02% |
| WP-1_6_3 | 46,779,350 | 46,779,346 | 6,900,299,084 | 45.48% | 99.37% | 98.03% |
| WP-2_2_1 | 45,431,620 | 45,431,618 | 6,696,766,024 | 44.40% | 99.35% | 97.98% |
| WP-2_2_2 | 41,444,462 | 41,444,462 | 6,118,822,356 | 45.02% | 99.33% | 97.94% |
| WP-2_2_3 | 44,847,832 | 44,847,830 | 6,647,078,696 | 44.64% | 99.32% | 97.90% |
| WP-2_4_1 | 47,320,236 | 47,320,232 | 6,995,702,218 | 44.77% | 99.31% | 97.89% |
| WP-2_4_2 | 50,495,718 | 50,495,718 | 7,426,382,380 | 44.85% | 99.40% | 98.15% |
| WP-2_4_3 | 38,149,254 | 38,149,254 | 5,668,951,744 | 44.97% | 99.25% | 97.67% |
| WP-2_6_1 | 45,875,408 | 45,875,408 | 6,809,305,152 | 45.50% | 99.32% | 97.87% |
| WP-2_6_2 | 38,223,388 | 38,223,388 | 5,672,126,028 | 45.46% | 99.22% | 97.55% |
| WP-2_6_3 | 43,145,864 | 43,145,862 | 6,410,919,064 | 45.15% | 99.34% | 97.94% |
| WP-3_2_1 | 56,895,334 | 56,895,334 | 8,386,150,190 | 45.07% | 99.36% | 98.01% |
| WP-3_2_2 | 40,010,126 | 40,010,124 | 5,949,249,904 | 44.82% | 99.24% | 97.64% |
| WP-3_2_3 | 55,646,620 | 55,646,616 | 8,188,815,308 | 45.18% | 99.36% | 98.00% |
| WP-3_4_1 | 48,826,146 | 48,826,144 | 7,247,616,754 | 45.23% | 99.35% | 97.94% |
| WP-3_4_2 | 51,766,740 | 51,766,740 | 7,653,381,816 | 44.95% | 99.35% | 97.97% |
| WP-3_4_3 | 47,163,608 | 47,163,608 | 6,996,857,978 | 45.66% | 99.29% | 97.78% |
| WP-3_6_1 | 37,472,582 | 37,472,582 | 5,572,472,500 | 46.20% | 99.28% | 97.73% |
| WP-3_6_2 | 43,224,934 | 43,224,934 | 6,427,003,588 | 45.53% | 99.26% | 97.71% |
| WP-3_6_3 | 44,442,548 | 44,442,546 | 6,594,769,894 | 45.51% | 99.34% | 97.96% |

**Table S2 Statistical results of alignment rate between reads and reference sequence**

| Sample | Unmapped  Reads(%) | Mapped  Reads(%) | Secondary  alignments(%) | Unique  alignments(%) |
| --- | --- | --- | --- | --- |
| WP_2_1 | 8,112,988(14.79) | 46,749,466(85.21) | 1,882,380(3.43) | 44,867,086(81.78) |
| WP_2_2 | 7,540,256(15.81) | 40,142,030(84.19) | 1,520,725(3.19) | 38,621,305(81.0) |
| WP_2_3 | 7,708,149(15.95) | 40,608,767(84.05) | 1,463,694(3.03) | 39,145,073(81.02) |
| WP_4_1 | 6,144,278(13.84) | 38,236,074(86.16) | 1,337,024(3.01) | 36,899,050(83.14) |
| WP_4_2 | 6,737,230(13.49) | 43,215,026(86.51) | 1,310,212(2.62) | 41,904,814(83.89) |
| WP_4_3 | 5,819,742(11.82) | 43,418,876(88.18) | 5,762,707(11.7) | 37,656,169(76.48) |
| WP_6_1 | 6,897,244(13.71) | 43,398,932(86.29) | 1,459,503(2.9) | 41,939,429(83.38) |
| WP_6_2 | 5,371,954(12.83) | 36,494,718(87.17) | 929,994(2.22) | 35,564,724(84.95) |
| WP_6_3 | 6,202,688(12.82) | 42,171,332(87.18) | 1,176,219(2.43) | 40,995,113(84.75) |
| WP-1_2_1 | 7,524,826(14.71) | 43,639,934(85.29) | 1,604,083(3.14) | 42,035,851(82.16) |
| WP-1_2_2 | 5,110,928(12.24) | 36,658,852(87.76) | 6,850,513(16.4) | 29,808,339(71.36) |
| WP-1_2_3 | 5,080,689(11.99) | 37,305,805(88.01) | 12,231,672(28.86) | 25,074,133(59.16) |
| WP-1_4_1 | 5,269,766(13.35) | 34,197,662(86.65) | 891,478(2.26) | 33,306,184(84.39) |
| WP-1_4_2 | 6,004,892(14.66) | 34,951,718(85.34) | 1,888,323(4.61) | 33,063,395(80.73) |
| WP-1_4_3 | 6,515,079(13.91) | 40,333,743(86.09) | 1,122,282(2.4) | 39,211,461(83.7) |
| WP-1_6_1 | 4,754,634(12.25) | 34,052,016(87.75) | 1,535,349(3.96) | 32,516,667(83.79) |
| WP-1_6_2 | 5,611,435(12.91) | 37,840,943(87.09) | 1,077,557(2.48) | 36,763,386(84.61) |
| WP-1_6_3 | 6,092,748(13.02) | 40,686,598(86.98) | 2,082,297(4.45) | 38,604,301(82.52) |
| WP-2_2_1 | 6,979,589(15.36) | 38,452,029(84.64) | 1,071,056(2.36) | 37,380,973(82.28) |
| WP-2_2_2 | 5,650,990(13.64) | 35,793,472(86.36) | 1,050,792(2.54) | 34,742,680(83.83) |
| WP-2_2_3 | 6,656,600(14.84) | 38,191,230(85.16) | 1,658,620(3.7) | 36,532,610(81.46) |
| WP-2_4_1 | 6,648,549(14.05) | 40,671,683(85.95) | 1,110,398(2.35) | 39,561,285(83.6) |
| WP-2_4_2 | 7,257,870(14.37) | 43,237,848(85.63) | 1,221,219(2.42) | 42,016,629(83.21) |
| WP-2_4_3 | 5,114,276(13.41) | 33,034,978(86.59) | 862,858(2.26) | 32,172,120(84.33) |
| WP-2_6_1 | 5,699,637(12.42) | 40,175,771(87.58) | 1,181,826(2.58) | 38,993,945(85.0) |
| WP-2_6_2 | 4,521,530(11.83) | 33,701,858(88.17) | 887,839(2.32) | 32,814,019(85.85) |
| WP-2_6_3 | 5,579,638(12.93) | 37,566,224(87.07) | 1,029,056(2.39) | 36,537,168(84.68) |
| WP-3_2_1 | 7,589,333(13.34) | 49,306,001(86.66) | 1,641,204(2.88) | 47,664,797(83.78) |
| WP-3_2_2 | 5,726,270(14.31) | 34,283,854(85.69) | 1,407,056(3.52) | 32,876,798(82.17) |
| WP-3_2_3 | 7,344,860(13.2) | 48,301,756(86.8) | 2,047,918(3.68) | 46,253,838(83.12) |
| WP-3_4_1 | 6,540,176(13.39) | 42,285,968(86.61) | 1,711,598(3.51) | 40,574,370(83.1) |
| WP-3_4_2 | 7,304,312(14.11) | 44,462,428(85.89) | 1,756,317(3.39) | 42,706,111(82.5) |
| WP-3_4_3 | 6,219,440(13.19) | 40,944,168(86.81) | 1,613,399(3.42) | 39,330,769(83.39) |
| WP-3_6_1 | 4,413,077(11.78) | 33,059,505(88.22) | 1,696,238(4.53) | 31,363,267(83.7) |
| WP-3_6_2 | 5,504,268(12.73) | 37,720,666(87.27) | 1,377,547(3.19) | 36,343,119(84.08) |
| WP-3_6_3 | 5,813,666(13.08) | 38,628,880(86.92) | 1,644,593(3.7) | 36,984,287(83.22) |

**Table S3 Primers used for qRT-PCR in this study**

| Primer name | Fequence (5’-3’) |
| --- | --- |
| CmoCh02G017160-F | TCGGCTCGCAATCGCTAATA |
| CmoCh02G017160-R | TTTCAAGGCAAGTGGCTCAATA |
| CmoCh01G019880-F | GCTGCCTGAAGTTTCCGTGTT |
| CmoCh01G019880-R | ACCTTCGACAGCCAATGGAGAC |
| CmoCh08G007820-F | GGCTTCACTCCACAGATTTCTTG |
| CmoCh08G007820-R | GCTGAGCTGCTTCCACTTGC |
| CmoCh08G002930-F | AGCGGCGTATGCGTTCAGA |
| CmoCh08G002930-R | GCTCTTCCAAAACCTCATTATCCA |
| CmoCh04G002030-F | GTGCCTGCAAGTTTCTTAGACGA |
| CmoCh04G002030-R | GCACCAAACACTTTATGAACAGCA |
| CmoCh18G001780-F | AACGTGGAGCCGAAGAAGGA |
| CmoCh18G001780-R | GAGTGGTCGCAGATGGAGGAGT |
| CmoCh06G006570-F | ACAAAGGCGAGGAGAATAAAGG |
| CmoCh06G006570-R | ACGACTGAGTCATCGAAACCAA |
| CmoCh01G005840-F | ACCTCCAAATATCGTCCGCCT |
| CmoCh01G005840-R | TGGCAATCTTCTTAACCGCAAC |
